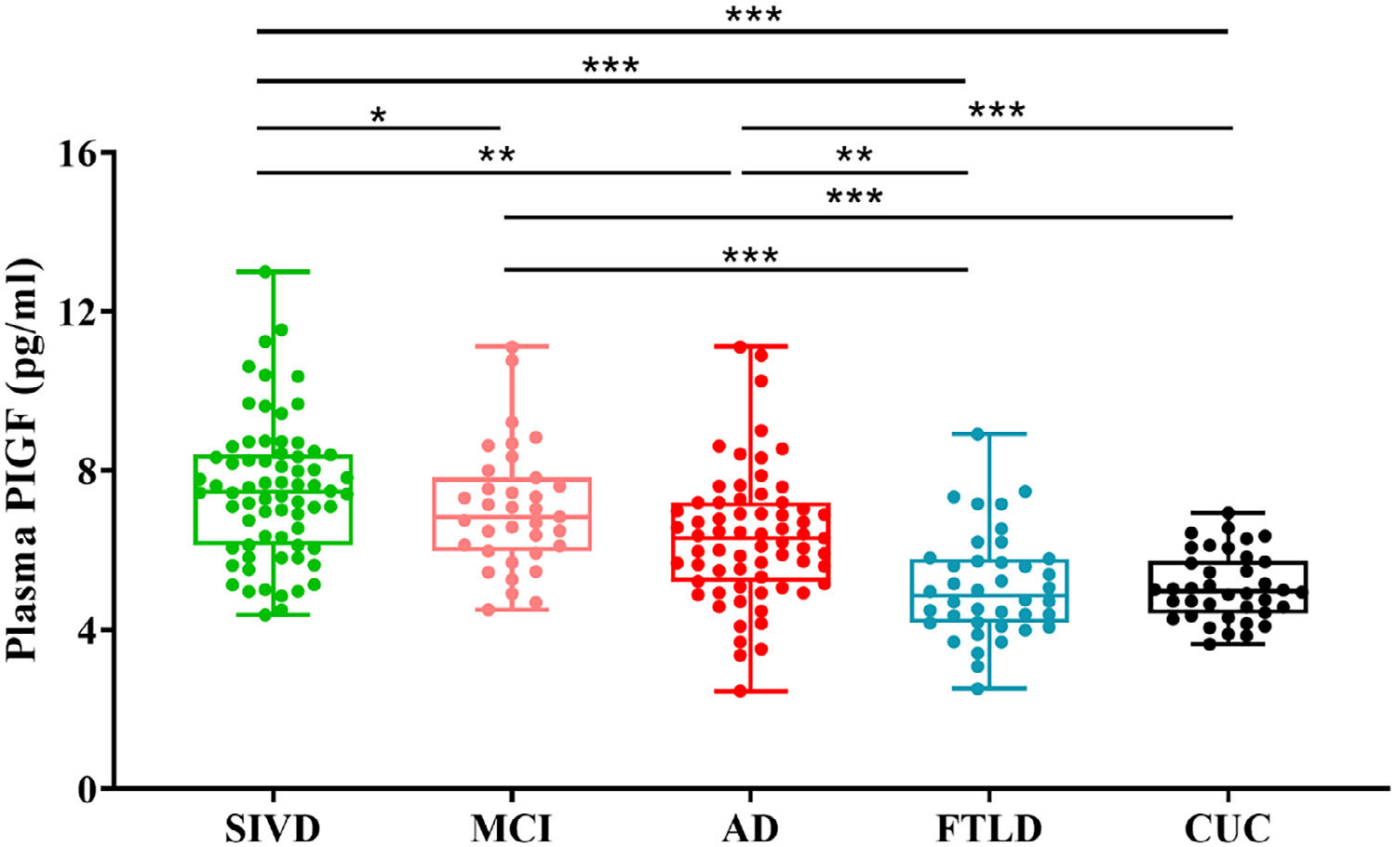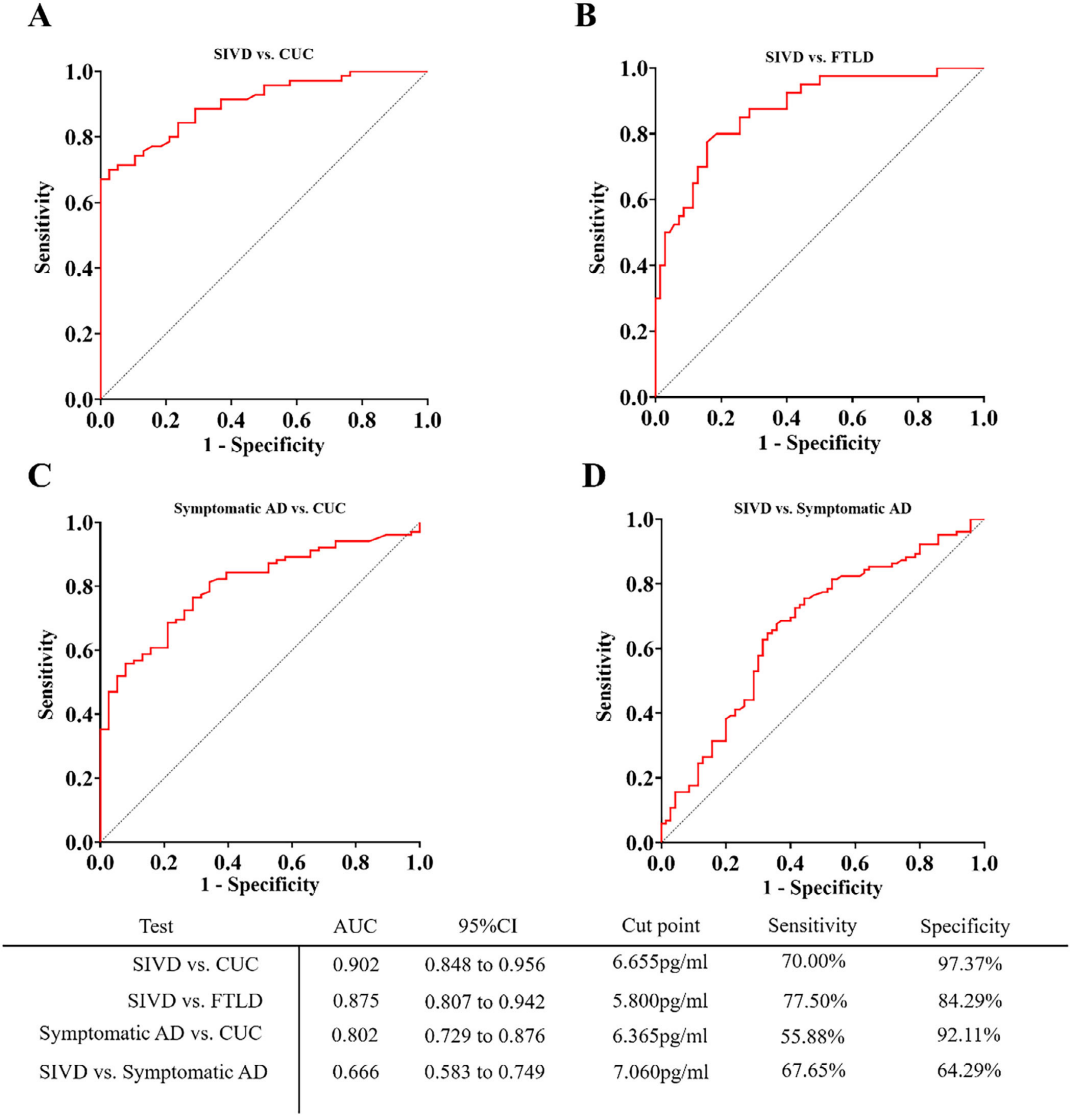# Plasma placental growth factor is a biomarker for subcortical ischemic vascular dementia

**DOI:** 10.1002/alz70856_099609

**Published:** 2025-12-24

**Authors:** Ping Che, Nan Zhang

**Affiliations:** ^1^ Tianjin Medical University General Hospital, Jin Tian, Jin Tian, China; ^2^ Department of Neurology, Tianjin Medical University General Hospital, Jin Tian, Jin Tian, China

## Abstract

**Background:**

Cerebral small vessel disease (SVD) played a pivotal role in the progression of subcortical ischemic vascular dementia (SIVD), a major subtype of vascular dementia. Placental growth factor (PlGF) was an angiogenic protein belonging to the vascular endothelial growth factor family, and may regulated cerebrovascular permeability. We aimed to detected its performance as a potential diagnostic biomarker for SIVD, and further explored its correlations cognitive function.

**Method:**

Seventy patients with SIVD, 102 patients with symptomatic Alzheimer's disease (AD), 40 patients with frontotemporal dementia (FTLD), and 38 cognitively unimpaired controls (CUCs) were included. All participants underwent clinical evaluation, cognitive testing and magnetic resonance imaging (MRI) scan. Additionally, the plasma concentration of PlGF was detected using an electrochemiluminescence immunoassay.

**Result:**

Plasma PlGF concentrations were significantly elevated in patients with SIVD (7.51±1.74 pg/ml), and showed high accuracies for discriminating patients with SIVD from CUCs (area under the curve [AUC] = 0.902) and FTLD patients (AUC = 0.875), but weak for patients with symptomatic AD (AUC = 0.666). In addition, plasma PlGF levels were negatively correlated with scores of memory (*r* = ‐0.334, *P* < 0.001), information processing (*r* = ‐0.263, *P* < 0.001), executive function (*r* = ‐0.285, *P* < 0.001), language (*r* = ‐0.178, *P* = 0.006), visuospatial function (*r* = ‐0.304, *P* < 0.001), and total cognitive function (*r* = ‐0.338, *P* < 0.001). These correlations still survived after adjusting for age, sex, and education.

**Conclusion:**

Plasma PlGF functions as a potential diagnostic biomarker for SIVD patients.